# The microbiota of hematophagous ectoparasites collected from migratory birds

**DOI:** 10.1371/journal.pone.0202270

**Published:** 2018-08-27

**Authors:** Francesco Cerutti, Paola Modesto, Francesca Rizzo, Alessandra Cravero, Irena Jurman, Stefano Costa, Mauro Giammarino, Maria Lucia Mandola, Mariella Goria, Slobodanka Radovic, Federica Cattonaro, Pier Luigi Acutis, Simone Peletto

**Affiliations:** 1 S.S. Genetica e Immunobiochimica, Istituto Zooprofilattico Sperimentale del Piemonte, Liguria e Valle d'Aosta, Torino, Italy; 2 S.S. Sezione di Genova, Istituto Zooprofilattico Sperimentale del Piemonte, Liguria e Valle d'Aosta, Genova, Italy; 3 S.S. Laboratorio Specialistico Diagnostica Molecolare Virologica e Ovocoltura, Istituto Zooprofilattico Sperimentale del Piemonte, Liguria e Valle d'Aosta, Torino, Italy; 4 S.S. Microbiologia Molecolare e Analisi Genomiche, Istituto Zooprofilattico Sperimentale del Piemonte, Liguria e Valle d'Aosta, Torino, Italy; 5 IGA Technology Services, Udine, Italy; 6 Laboratorio Chimico Camera Commercio Torino, Torino, Italy; 7 Department of Prevention, ASL CN 1, Racconigi (CN), Italy; University of Kentucky College of Medicine, UNITED STATES

## Abstract

Arthropod vectors are responsible for the transmission of human pathogens worldwide. Several arthropod species are bird ectoparasites, however, no study to date has characterized their microbiota as a whole. We sampled hematophagous ectoparasites that feed on migratory birds and performed 16S rRNA gene metabarcoding to characterize their microbial community. A total of 194 ectoparasites were collected from 115 avian hosts and classified into three groups: a) *Hippoboscidae* diptera; b) ticks; c) other arthropods. Metabarcoding showed that endosymbionts were the most abundant genera of the microbial community, including *Wolbachia* for *Hippoboscidae* diptera, Candidatus *Midichloria* for ticks, *Wolbachia* and *Arsenophonus* for the other arthropod group. Genera including pathogenic species were: *Rickettsia*, *Borrelia*, *Coxiella*, *Francisella*, *Bartonella*, *Anaplasma*. Co-infection with *Borrelia*-*Rickettsia* and *Anaplasma*-*Rickettsia* was also observed. A global overview of the microbiota of ectoparasites sampled from migratory birds was obtained with the use of 16S rRNA gene metabarcoding. A novel finding is the first identification of *Rickettsia* in the common swift louse fly, *Crataerina pallida*. Given their possible interaction with pathogenic viruses and bacteria, the presence of endosymbionts in arthropods merits attention. Finally, molecular characterization of genera, including both pathogenic and symbiont species, plays a pivotal role in the design of targeted molecular diagnostics.

## Introduction

Arthropod vectors are responsible of numerous diseases (named vector-borne diseases) worldwide [1]. Mosquitoes, ticks, Phlebotominae and Simuliidae flies are ectoparasites that can transmit viruses (e.g., Dengue virus, Yellow fever virus, West Nile virus (WNV), and Zika virus), bacteria (e.g., *Borrelia* spp., *Rickettsia* spp., *Francisella tularensis*, *Coxiella burnetii*), and parasites (e.g., malaria *Plasmodium spp*., trypanosomes, *Leishmania spp*.) [1]. The 2016 Zika virus pandemic is only the most recent example of a global vector-borne disease emergency among the many other pathogens for which there is an epidemic trend [2]. For example, the hard tick *Ixodes ricinus*, present throughout Europe, is involved in the transmission of a variety of pathogens of medical and veterinary importance including *Borrelia burgdorferi* s.l., tick-borne encephalitis virus, *Anaplasma phagocytophilum*, *Francisella tularensis*, *Rickettsia helvetica* and *Rickettsia monacensis*, *Babesia divergens* and *Babesia microti*, Louping ill virus, and Tribec virus [3].

Some of the arthropods responsible for disease transmission share their environment with birds. Mosquitoes belonging to *Culex* are, in fact, mainly ornithophilic and are the main vectors of WNV and Usutu virus. Moreover, birds physically carrying arthropods (such as ticks or mites) feeding on them can introduce novel species to Europe, as recently recorded for the U.K. [4–6].

Owing to its geographical location, the Italian peninsula is crossed by migratory routes from North and sub-Saharan Africa. To our knowledge, no data have been published on the whole microbiota of ectoparasites collected directly from migratory birds, though a few studies have described the presence and prevalence of specific genera of bacteria in ticks collected from birds or their nests [7, 8]. Since these ectoparasitic arthropods may carry pathogens, it may be relevant to study their microbial communities. Other than bacteria of public health interest, the microbiota of arthropods is complex. It has been described in ticks and mosquitoes [9–12] and the role of symbionts in influencing the microbial composition has been highlighted mainly in its interaction with pathogens. Symbionts like *Wolbachia* can influence arthropod reproduction, including male-killing, parthenogenesis, feminization, and embryonic mortality [13]. Furthermore, they may evolve the necessary adaptations to parasitize vertebrate cells, as recently demonstrated that the intracellular bacterium *Coxiella burnetii* evolved from a maternally-inherited endosymbiont of ticks [14]. Adaptation may also occur in the opposite direction, as in the case of the *Francisella*-like endosymbiont that evolved from *Francisella tularensis* [15].

For this study, we collected ectoparasites feeding on migratory birds during ringing sessions and then processed the arthropod samples for 16S rRNA gene metabarcoding to characterize their microbial community. Special care was paid to identify the genera commonly associated with pathogens. The samples reporting these bacteria were further tested with genus-specific or species-specific molecular assays.

## Materials and methods

### Sample collection

The ectoparasites were collected from birds during ringing sessions from November 2012 to October 2014. A total of 35 sessions were carried out at 14 different sites in five regions (Piedmont, n = 105; Lombardy, n = 5; Sicily, n = 4; Latium, n = 1; Liguria, n = 1; S1 Fig). A total of 194 ectoparasites were collected from 115 birds, divided into 120 pools by parasite type [a) *Hippoboscidae* diptera; b) ticks; c) other arthropods], host species, sampling site, date, and location. The species included in the other-arthropods group were: *Anatoecus dentatus*, *Anaticola*, *Lucilia caesar*, *Colpocephalum turbinatum*, *Anystis*, and *Aphidiinae* spp. Details on host species are reported in S1 Table.

The birds were caught with mist nets according to the Euring Ringing System and retrieved by authorized personnel. After capture the birds were ringed with a metal ring on the right leg. In Italy, bird leg rings are supplied by the Institute for Environmental Protection and Research (ISPRA) and they bear a unique, permanent code identifying any ringed bird for life. The birds were then identified by species, sexed, and assigned to age categories according to plumage. They were released after ectoparasite collection by veterinarians with ISPRA authorization. Being a non-invasive procedure, no special permission was needed for collection. Parasites from common swifts, mainly *Hippoboscidae* diptera, were collected either directly from the birds or from their nests in dedicated stations.

To preserve nucleic acids and obtain good quality material for metabarcoding, live parasites were stocked in RNAlater^TM^ stabilization solution (Invitrogen, Carlsbad, CA, USA) and stored at -80°C until processed. The parasites collected from each bird were pooled together in a single vial, except for two birds (*Apus apus*), for which the parasites were stored separately for preliminary evaluation of RNA integrity. Data on sampling site location, bird age, sex, and health status were collected and entered in a database.

### RNA extraction

As the rationale of the study was to describe the living bacteria (i.e., synthesizing RNA), we analyzed the total RNA to characterize only the active microbiota and to remove bias from the DNA carried over from dead prokaryotic cells. RNA purification was performed with TRIzol™ (Invitrogen) in combination with a Nucleospin miRNA kit (Macherey-Nagel, Düren, Germany) following the manufacturer’s protocol for RNA purification of small and large RNA in two fractions. The large and small RNA fractions were stored at -80°C for further analysis.

Total RNA concentration and purity was estimated using a spectrophotometer for small volumes (Vivaspec, Sartorius, Göttingen, Germany) and a fluorometer (Qubit 2.0, Thermo Fisher Scientific, Waltham, MA, USA). The quality of total RNA was evaluated using a 2100 BioAnalyzer and an RNA 6000 Nano Kit (Agilent Technologies, Santa Clara, CA, USA). Though it was not possible to calculate the RIN (RNA Integrity Number) values [16], since the 28S rRNA subunit of many arthropods contains two hydrogen-bonded fragments that dissociate and co-migrate with the 18S subunit [17], the graph showed a 28S/18S sharp peak associated with a flat baseline that indicated the absence of degradation.

### Reverse transcription and arthropod species identification

Total RNA was reverse transcribed using a High-Capacity cDNA Reverse Transcription Kit (Thermo Fisher Scientific) with 7 μl RNA as input and then stored at -20°C until processed. Vector species was determined by partial amplification and sequencing of the cytochrome c oxidase I (COI) gene, as described by Hebert and colleagues [18]. Briefly, the reaction mix was composed of 12.5 μl SuperMix PCR-UDG 2X (qPCR ProbesMaster, Jena Bioscience, Jena, Germany), 0.38 μl primer LC01490 20 μM, 0.38 μl primer HC02198 20 μM, 11 μl H2O, 0.75 μl cDNA, for a total volume of 20 μl. The thermal profile was: 50°C x 2 m; 95°C x 2 m; 40 cycles {94°C x 30 s, 49°C x 30 s, 72°C x 1 m}; 72°C x 5 m.

Successful amplification was verified using E-Gel® precast agarose gels at 2% (Thermo Fisher Scientific). Amplicons were then purified with a EUROGOLD Cycle-Pure kit (Euroclone, Pero, MI, Italy). The cycling reaction was performed with a BigDye® Terminator v1.1 cycle sequencing kit (Thermo Fisher Scientific): 2 μl BigDye® Terminator v1.1 ready reaction mix, 1 μl 5X sequencing buffer, 0.32 μl primer 100 μM, 4.68 μl H2O, 2 μl purified amplicon. The thermal profile was: 96°C x 1 m; 25 cycles at 96°C x 1 m, 50°C x 5 s, 60°C x 4 m. The reaction was purified with a GE Healthcare Illustra™ AutoSeq G-50 columns kit (GE Healthcare, Chicago, IL, USA) to remove dye terminators, and then submitted to sequencing on an Applied Biosystems AbiPrism 3130 (Foster City, CA, USA).

Chromatograms were analyzed with Sequencing Analysis v5.2 software (Thermo Fisher Scientific) for the base call and with FinchTV (Geospiza, Inc, Seattle, WA, USA) for hand editing. The obtained nucleotide sequences were used as query in a Blastn search on the GenBank nt database and in the BOLD database (Barcoding Of Life Database, www.boldsystems.org).

### 16S metabarcoding

The 16S rRNA gene metabarcoding of 116 out of 120 samples was performed following the protocol suggested by Illumina (four samples were discarded as they were not of adequate quality for sequencing). Briefly, 22.5 ng of cDNA was used as input for the first PCR using 16S amplicon PCR forward and reverse primers, amplifying V3-V4 regions of the 16S rDNA. After purification and second (index) PCR with a Nextera XT Index kit (Illumina, San Diego, CA, USA), the libraries were normalized according to fragment length and dsDNA molarity. The samples were pooled and processed in four sessions on a MiSeq platform (Illumina) using a MiSeq reagent kit v3-600 for 2x300 paired-end sequencing at the IGA Technology Services facility. The datasets generated and analyzed for this study are available in the BioProject database, with SubmissionID: SUB2898018 and BioProject ID: PRJNA396024.

### Bioinformatic and statistical analyses

A first level analysis for all samples was achieved with MiSeq Reporter Metagenomics Workflow (MSR, Illumina) to gain an overview of the microbial community for each pool. The dataset was then analyzed following DADA2 workflow within the R framework, including quality check, error rate estimation, forward/reverse reads merge, chimera removal, ribosomal sequence variants (RSVs, equivalent to OTUs) determination, and taxa assignment to the GreenGenes gg_13_8_train_set_97, RDP Training Set 14 and SILVA version 128 reference databases for comparison [19–23]. Since SILVA performed better than the other two in classifying arthropod bacterial symbionts to appropriate taxa, only these results are presented. Data were also analyzed with the QIIME v.1.9.1 pipeline, and the results were largely consistent with those obtained with DADA2.

Alpha and beta diversities were estimated with the *pyhloseq* and *vegan* packages and visualized with the *ggplot2* package, and differential expression was assessed with *DeSeq2* [24–27]. Alpha diversity was estimated based on the observed species and Shannon index using the whole dataset after removing RSVs unassigned or assigned to Eukaryota. Beta diversity was estimated based on evenly sampled Bray-Curtis distance after filtering the low-frequency RSVs, pruning the samples with a sample size less than 1,800 and rarefaction to sample size of 1,800, where sample size was the number of individuals observed for each sample. Only data from DADA2 analysis using the SILVA database are presented in Results.

### Molecular diagnostics for potential pathogen confirmation

To confirm the presence of the prokariotic genera identified by 16S rRNA metabarcoding, the pools were tested using a genus-specific or species-specific PCR for each of the potential pathogens detected by the MiSeq Reporter Metagenomics Workflow. The molecular tests included the following genera: *Rickettsia* spp., *Anaplasma* spp., *Borrelia* spp., *Coxiella* spp., *Francisella* spp., and *Bartonella* spp. For all PCR assays, amplicons were purified and submitted to Sanger sequencing as described earlier for the PCR targeting the COI gene.

Two different PCR assays to confirm and characterize *Rickettsia* were used: one targeting the citrate synthase gene, according to Regnery and colleagues [28], and the other targeting the 16S rRNA gene, according to Sprong and colleagues [29]. A PCR amplifying the partial 16S rRNA gene was used to identify *A*. *phagocytophilum* according to Stuen and colleagues [30]. Two different approaches were applied to identify *Borrelia burgdorferi* s. l. Real-time PCR targeting a tract of the 23S rRNA gene, highly conserved in all *Borrelia* species, was used to confirm positivity, as described in Courtney and colleagues [31]. An end-point PCR targeting a fragment of the flagellin gene, specific for the *Borrelia burgdorferi* s. l. group, was then used to determine whether the detected strain belonged to the causative agents of Lyme borreliosis [32]. The genomic group of samples positive for *Borrelia burgdorferi* s. l. was then identified by sequencing. A qualitative PCR targeting the IS1111 repetitive transposon-like region of *Coxiella burnetii* was performed to confirm *Coxiella* spp., as recommended by the Manual of Diagnostic Tests and Vaccines for Terrestrial Animals [33, 34]. *Francisella* spp. was investigated using a real-time PCR TaqMan® *Francisella tularensis* detection kit (Applied Biosystems) that targets the Tul4 and fopA genes. *Francisella* characterization was performed by targeting the 16S rRNA gene, according to Forsman and colleagues [35].

## Results

### Host and parasite species

A total of 115 bird hosts were included. S1 Table presents the species and the number of samples collected. Hosts were classified based on their migratory behavior as: resident, short-distance, mid-distance (North Africa) or long-distance (trans-Sahara) migration. A total of 194 parasites were collected and categorized into three groups based on the type of arthropod: *Hippoboscidae* diptera (n = 51, pool (n) = 49), ticks (n = 114, pool (n) = 60), other arthropods (OA, n = 29, pool (n) = 7). *Hippoboscidae* diptera were collected from common swifts (*Apus apus*) and classified by barcoding as *Crataerina pallida*. One sample of *Hippoboscidae* was collected from a goldcrest (*Regulus regulus*) and was classified as *Ornithomya fringillina*. The tick group included species of the genera *Ixodes*, *Hyalomma*, *Ambylomma*, and *Haemaphysalis*. The OA included lice (Mallophaga: *Colpocephalum turbinatum*, *Anatoecus dentatus*, and unidentified species), blowflies (Diptera: Calliphoridae: *Lucilia caesar*), mites (Trombidiformes: *Anystis*), the parasitoid wasps (Hymenoptera: Braconidae: Aphidiinae). A detailed list of ectoparasite species and number of sampled individuals and pools is given in S2 Table.

### 16S metabarcoding

**Fig 1:**
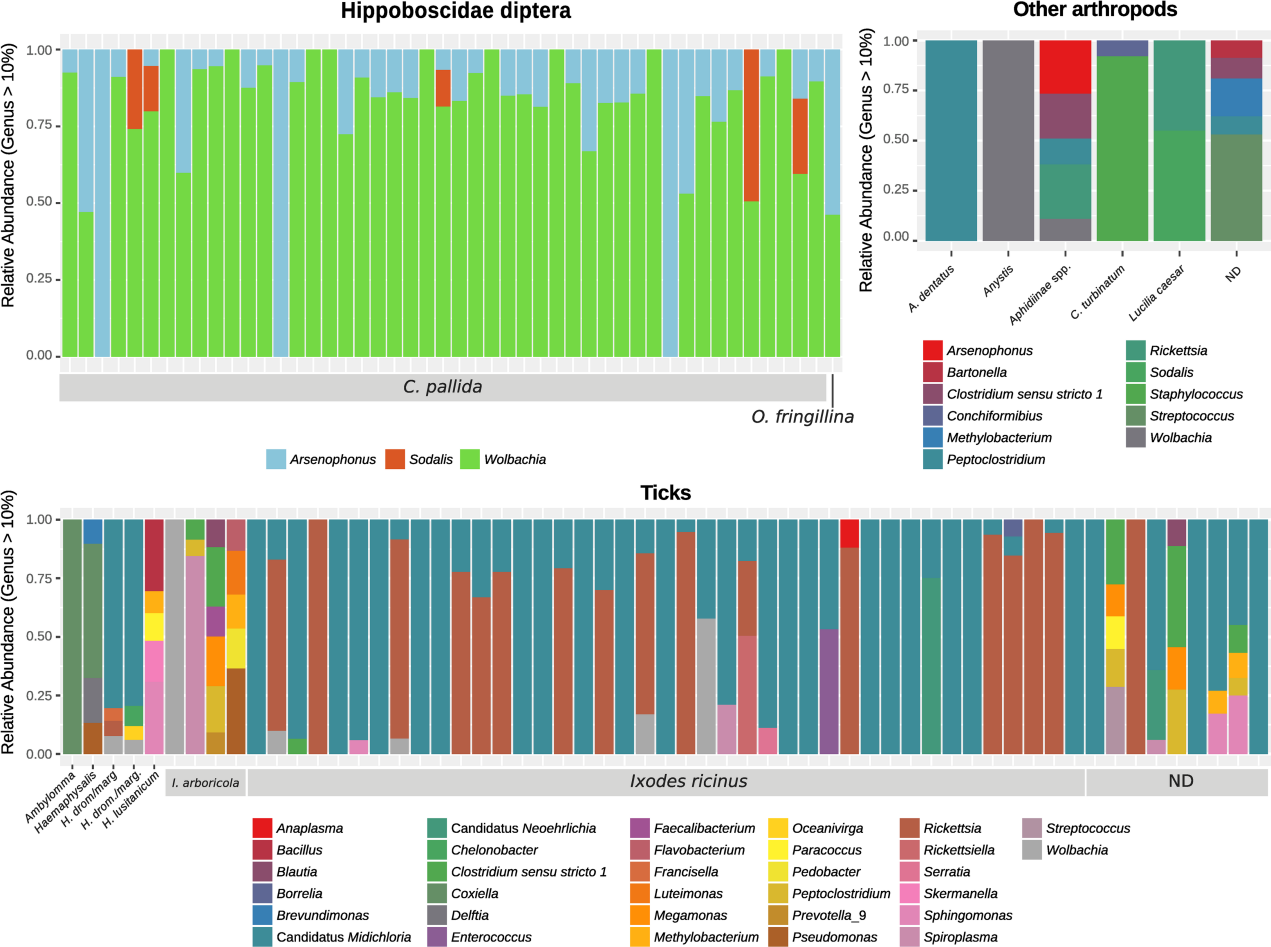
Genus composition accounting for 90% abundance for each sample. Samples are grouped by type of parasite (*Hippoboscidae* diptera, OA, and Ticks).

A total of 116 pools were processed for V3-V4 16S rRNA gene amplification and massive parallel sequencing on an Illumina MiSeq platform, generating also a taxonomic report with MiSeq Reporter Software. The total amount of reads generated was 45,286,016 (median = 323,508, Q1 = 203,740, Q3 = 510,136); after quality filtering 24,012,404 reads per pair (median = 82,992, Q1 = 48,206, Q3 = 143,460, with read length uniformed to 230 bp) were obtained. Details on raw and filtered read numbers for each sample are reported in S3 Table. The resulting 257,298 dereplicated non-chimeric sequences were assigned to 2,257 RSVs, belonging to Bacteria and Eukaryota domains. Taxa assigned to Eukaryota or unassigned were removed, reducing the final number of RSVs to 2,184, classified in 23 different phyla. The most abundant phyla in the whole dataset were the Proteobacteria (90.72%) and the Firmicutes (5.44%). The most common genera for the whole dataset are reported in Table 1, and the taxa with relative abundance >1% are reported by group in Table 2. Fig 1 presents a graphical overview of the genera identified in each sample. The most abundant taxonomic groups of the bacterial composition at a lower taxonomical scale included symbionts like *Wolbachia*, *Arsenophonus*, and Candidatus *Midichloria mitochondrii*.

**Table 1:** Most abundantgenera (%) in the whole data set.

Genus	Relative abundance (%)
*Wolbachia*	35.79
Candidatus_*Midichloria*	25.45
*Rickettsia*	12.57
*Arsenophonus*	8.19
*Sodalis*	1.60
*Coxiella*	1.22
*Clostridium_sensu_stricto_1*	0.96

**Table 2:** Composition of class, order, family, and genus (expressed as relative abundance, %) with >1% relative abundance reported for each sample group.

	Class		Order		Family		Genus	
***Hippoboscidae* Diptera**	*Alphaproteobacteria*	78.28	*Rickettsiales*	78.27	*Rickettsiaceae*	78.26	*Wolbachia*	78.23
	*Gammaproteobacteria*	21.69	*Enterobacteriales*	21.69	*Enterobacteriaceae*	21.69	*Candidatus Phlomobacter*	17.95
							*Sodalis*	2.61
**Ticks**	*Alphaproteobacteria*	79.77	*Rickettsiales*	76.35	*Midichloriaceae*	51.33	*Candidatus Midichloria*	52.81
	*Gammaproteobacteria*	5.03	*Clostridiales*	4.77	*Rickettsiaceae*	25.29	*Rickettsia*	23.27
	*Clostridia*	4.77	*Legionellales*	3.30	*Coxiellaceae*	3.15	*Clostridium*	2.52
	*Bacilli*	2.18	*Entomoplasmatales*	1.83	*Clostridiaceae*	2.58	*Coxiella*	2.36
	*Mollicutes*	1.85	*Lactobacillales*	1.41	*Anaplasmataceae*	1.88	*Wolbachia*	2.01
			*Sphingomonadales*	1.38	*Sphingomonadaceae*	1.30	*Candidatus Neoehrlichia*	1.68
			*Rhizobiales*	1.04	*Enterococcaceae*	0.96	*Sphingomonas*	1.08
							*Enterococcus*	0.96
**OA**	*Alphaproteobacteria*	42.52	*Rickettsiales*	37.82	*Rickettsiaceae*	37.77	*Wolbachia*	29.46
	*Gammaproteobacteria*	24.98	*Enterobacteriales*	24.15	*Enterobacteriaceae*	24.15	*Arsenophonus*	16.38
	*Bacilli*	17.31	*Bacillales*	11.03	*Staphylococcaceae*	10.80	*Staphylococcus*	10.79
	*Clostridia*	9.48	*Clostridiales*	9.48	*Clostridiaceae*	4.55	*Rickettsia*	8.32
	*Betaproteobacteria*	1.79	*Lactobacillales*	5.61	*Streptococcaceae*	4.30	*Sodalis*	8.15
	*Bacteroidia*	1.30	*Rhizobiales*	3.10	*Lachnospiraceae*	2.03	*Clostridium*	4.35
			*Neisseriales*	1.48	*Methylobacteriaceae*	1.53	*Streptococcus*	4.29
			*Bacteroidales*	1.30	*Neisseriaceae*	1.48	*Methylobacterium*	1.53
					*Veillonellaceae*	1.35	*Megamonas*	1.31

To better visualize the distribution of the symbiont genera in the *Hippoboscidae* diptera, a bar plot of the relative abundance within the family is reported in S2 Fig. Due to the large number of genera detected, *Rickettsiales* from ticks are reported in a bar plot (S3 Fig). To better represent the diversity of the symbionts within parasite species, the number of RSVs belonging to each genus are summarized in Table 3. According to the DADA2 developers, this algorithm in able to detect true biological sequence variants that might be considered different bacterial strains. The composition in RSVs of the major symbiont genera of *Hippoboscidae* diptera and ticks are reported in S4 Fig and S5 Fig.

**Table 3:** Number of ribosomal sequence variants (RSVs) for each of the main symbiont genera characterized in the entire data set.

Genus	No. of RSVs
*Arsenophonus*	7
*Sodalis*	4
*Wolbachia*	12
*Candidatus_Midichloria*	10
*Rickettsia*	7

### Bioinformatic and statistical analysis

**Fig 2:**
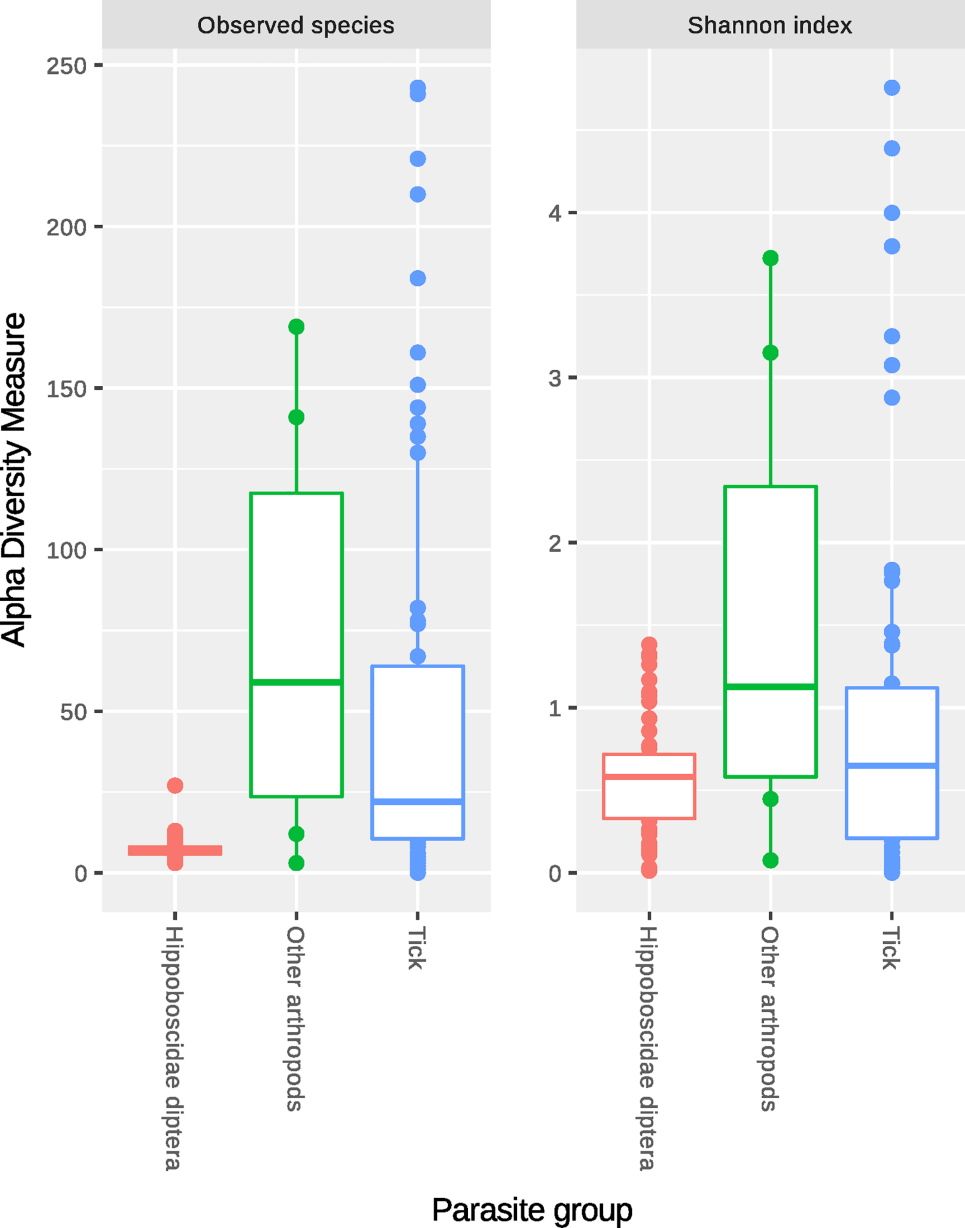
Box-plot of the main indexes for alpha diversity by parasite group. Indexes are observed species and Shannon index.

**Fig 3:**
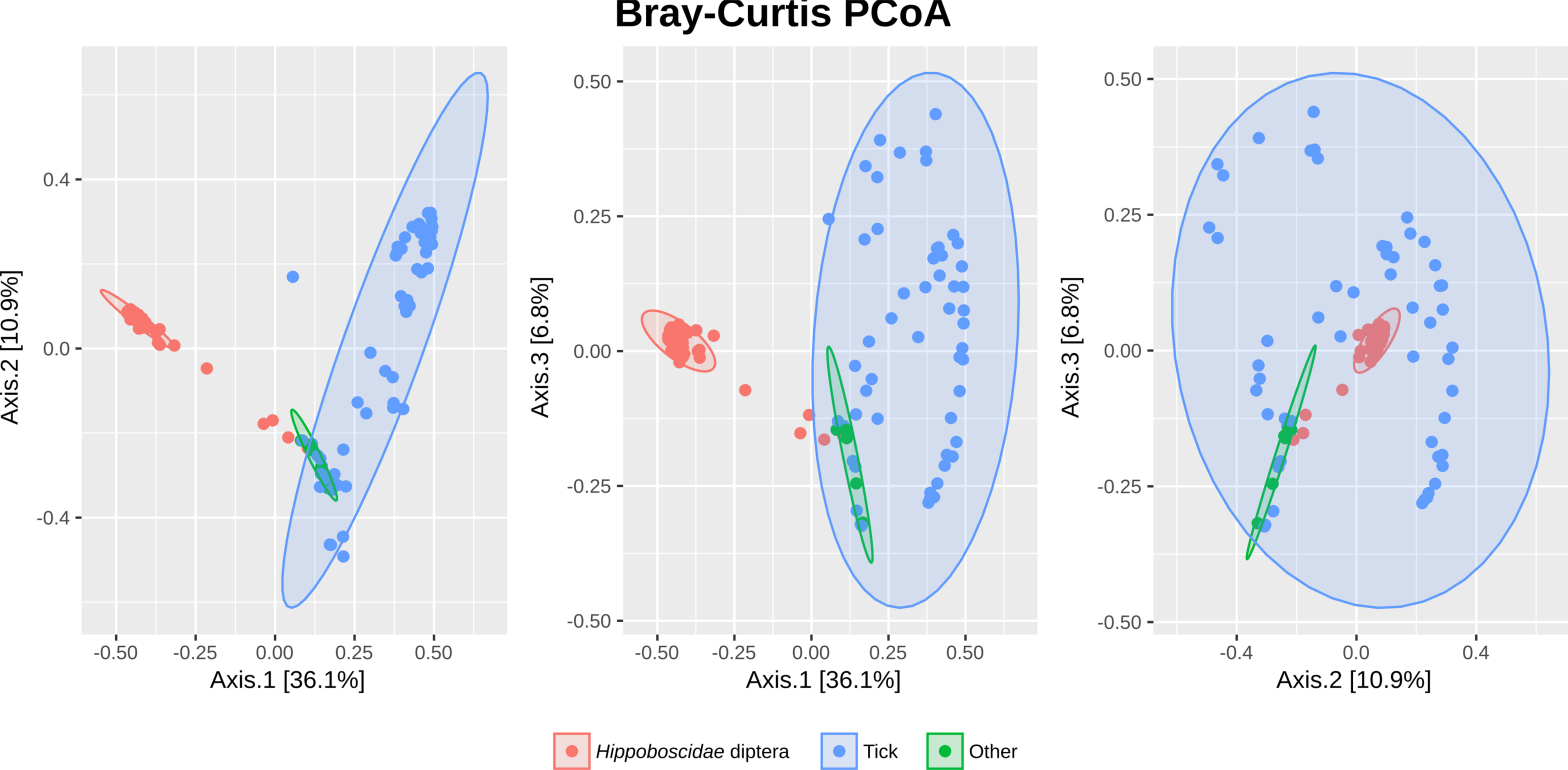
Principal coordinates analysis based on Bray-Curtis distances of the three separate groups. As reported by the axis label, the axis 1 shows 36.1% of variation in the samples.

The reports generated by the integrated pipeline of the Miseq Reporter were analyzed. Based on these results, the samples were considered positive or negative for the detected potentially pathogenic genera. Table 4 reports the number of samples in which the MSR found the corresponding genus (MSR Hits). The microbiota of the three groups of ectoparasites differed in microbial composition by both abundance and represented taxa. Box-plots illustrating alpha diversity are reported in Fig 2. The Shannon index showed no statistically significant difference between the groups (box-plot Fig 2). There was a statistically significant difference in the number of species for the three groups. The box-plot represents the low number of species in the *Hipposboscidae* diptera group as compared to the other two groups. The principal coordinates analysis based on the Bray-Curtis distance highlighted a difference in microbial composition in the microbiota of the *Hipposboscidae* diptera as compared to that of the ticks and the OA groups (PERMANOVA test implemented in the *adonis* function in *vegan*) (Fig 3).

**Table 4:** Hits and PCR positivity to genera including known pathogen species. Hits (OTUs matching a given genus) were obtained by MiSeq Reporter (MSR) analysis. Prevalence was calculated on 116 samples. The 95% confidence interval (CI) of the prevalence is reported in brackets.

Genus	MSR Hits	MSR Prevalence	PCR confirmations	PCR prevalence
***Rickettsia***	110	94.8% (88.94–97.84)	47	40.5% (32.02–49.62)
***Ehrlichia***	98	84.5% (76.71–90.04)	-	-
***Borrelia***	17	14.7% (9.26–22.32)	10	8.6% (4.59–15.31)
***Coxiella***	6	5.2% (2.16–11.06)	0	0%
***Francisella***	4	3.4% (1.06–8.82)	2	1.7% (0.09–6.46)
***Bartonella***	4	3.4% (1.06–8.82)	0	0%
***Anaplasma***	3	2.6% (0.55–7.66)	2	1.7% (0.09–6.46)

### Molecular diagnostics for potential pathogen confirmation

As summarized in Table 4, *Rickettsia* and *Ehrlichia* were highly represented among the samples, with more than 80% of the samples having reads corresponding to these two genera. The diagnostic PCR for *Rickettsia* spp. confirmed only 47 positive samples (42% of hits, 40.5% of total samples), further characterized as *R*. *aeschlimannii*, *R*. *helvetica*, and *R*. *monacensis*. Some of the sequences were similar to unclassified endosymbionts, 6 of which were were close to *R*. *bellii*, a species found only in the Americas (USA, Brazil, Argentina, Costa Rica, Colombia, El Salvador, Peru) [36–42]. As described for *C*. *burnetii* [43], identification of *Rickettsia* endosymbionts by means of a PCR used for pathogen detection in routine work shows that these species may interfere with the correct diagnosis of pathogenic rickettsial species.

*Borrelia* spp. were confirmed in 10 samples, four of which were further confirmed by the PCR specific for *Borrelia burgdorferi* s.l. and classified as *B*. *valaisiana*. The two samples positive for *Francisella* spp. were *Francisella*-like endosymbionts, while the two *Anaplasma* positive samples belonged to *A*. *phagocytophilum*. *Ehrlichia* required a different approach, since the MSR identified *E*. *ovina* in 98 samples, a species poorly described in the literature. On the basis of this unexpectedly high prevalence, and in contrast to only three sequences registered in the NCBI database, we suspected a misclassification issue, so we randomly chose one sample and retrieved the reads assigned to this species. A Blast search against the NCBI 16S prokariotic rRNA database was performed and the results were then plotted in MEtaGenome ANalyzer. The output is reported in S6 Fig The reads were classified as *Anaplasmataceae*, and at a lower taxonomic level as Candidatus *Midichloria mitocondrii*, *Anaplasma* spp., and *Wolbachia* spp. For this reason, the samples were not tested for *Ehrlichia* spp.

Among the PCR-confirmed samples, the following co-infections were observed: *Borrelia*-*Rickettsia* (n = 9), two of which occurred in individual samples, and *Anaplasma*-*Rickettsia* (n = 2). Considering only the ticks, the prevalence of confirmed *Rickettsia* spp. in the tick-only group was 60.32% (95% CI: 47.98–71.47), 15.87% (95% CI 8.86–26.81) for *Borrelia* spp., and 18.37% (95% CI 9.98–31.36) for *Ixodes*. *Rickettsia* spp. was present in numerous samples and detected in parasites collected from resident (n = 2), short-distance (n = 32), mid-distance (n = 2), and long-distance (n = 11) migratory birds. *Borrelia* spp. was detected only in ticks from short- and mid-distance migratory birds.

## Discussion

With this study we wanted to describe the microbiota of ectoparasites collected from migratory birds since they constitute a route of introduction for exotic vector-borne diseases. The parasites were divided into three groups based on taxonomical features and sample size: *Hippoboscidae* diptera, ticks, and other arthropods. The microbiota of the *Hippoboscidae* diptera was composed of a limited number of species, as expressed by the low value of the observed species. Considering the evenness (Shannon index), the diversity was comparable to the tick and the OA groups. The low number of species may be explained by the predominance of symbiont species among the most abundant genera observed, such as *Wolbachia*, *Arsenophonus*, and *Sodalis* (*Sodalis* endosymbionts were also detected in *Craterina melbae*) [44–47]. The difference in microbial composition by number and taxonomy of the RSVs in the three groups is supported by the significant difference in the alpha and beta diversity, suggesting that the bacterial communities are heavily influenced by the parasite they live with. Briefly, the most abundant symbionts were *Wolbachia*, *Rickettsia*, *Arsenophonus*, and C. *Midichloria*. They were closely associated with the type of arthopod: *Hippoboscidae* were mainly colonized by *Wolbachia* and *Arsenophonus* and ticks by *Rickettsia* and *C*. *Midichloria*.

Regarding the distribution of the main genera in the *Hippoboscidae*, the microbial population of all but three samples was almost totally composed of *Wolbachia*. The three *Wolbachia*-free samples were totally colonized by *Arsenophonus*, while *Sodalis* was present only together with other symbionts. Only in three samples *Wolbachia* was the unique genus, for a total of 6 individuals with a single symbiont. Although the relative abundance is based on the family, the majority of the *Hippoboscidae* was colonized by at least two symbiont genera (S2 Fig).

*Wolbachia* strains seemed to be closely connected to host species (S4 Fig); *C*. *pallida* was mainly colonized by one variant and *O*. *fringillina* by another. In contrast, *Arsenophonus* in *C*. *pallida* had a slight diversity (with RSVs similar by 99.30–99.53%), while in *O*. *fringillina* it was present only as one RSV (similar to other *Arsenophonus* symbionts of *Ornithomya* species). The high homogeneity of *Wolbachia* suggests that it may be an obligate symbiont vertically inherited by maternal lineage. Differently, the diversity of *Arsenophonus* within samples suggests that it may have been transmitted horizontally or by other ways. The high presence of *Wolbachia* define this genus as the predominant symbiont. While it might be an obligate symbiont within *C*. *pallida*, the presence of other genera suggests that they still play an important role in the survival of *Hippoboscidae*, but further data are needed.

To our knowledge, this report is the first identification of *Rickettsia bellii* and *R*. *monacensis* in the *Hippoboscidae C*. *pallida*. Strains of *R*. *bellii* have been reported only for the Americas; similar strains have been detected in Australia, Thailand, Réunion Island, and Japan [44–47]. Our report is the first identification of *R*. *bellii* in Italy and Europe. This finding raises the question as to whether *C*. *pallida* behaves as an accidental vector for rickettsiosis or, if not competent for transmission, whether it might play a role as a sentinel parasite for the spread of arthropod-borne pathogens.

In ticks, the endosymbiont Candidatus *Midichloria* accounted for half of the RSV abundance in the samples, followed by *Rickettsia* spp. When *Rickettsia* spp. was present, it had the highest prevalence in almost all samples; only in one sample, *Rickettsia* was present but the predominant genus was *Midichloria*. As reported elsewhere, *Wolbachia* symbionts in ticks are rare [48]. Unlike a recent study on ticks in France, our study noted no relevant presence of *Acinetobacter*, but we did observe co-infection with pathogens and symbionts in our samples [49]. In the samples with only one tick, we observed co-infection mainly between *Rickettsia* and *Midichloria*; also *Wolbachia*, *Rickettsiella*, *Neoehrlichia*, and *Spiroplasma* were present together with other symbionts. Candidatus *Midichloria* was present mainly in *Ixodes ricinus* ticks, where it was represented by a unique variant. Unfortunately, the sample size was too small to make further observations for other species like *I*. *arboricola* and *Hyalomma* spp.

Candidatus *Midichloria* was first described in 2006 as an endosymbiont of *Ixodes ricinus*, and was later also detected in other hard ticks (*Ixodidae*) in Italy [50–51]. The detection of circulating DNA and the presence of antibodies against an antigen against *M*. *mitochondrii* in humans and mammals suggest that it might represent a novel group of vector-borne agents [52, 53].

The role of endosymbionts in arthropods has been partially described and a strong correlation with pathogen replication and transmission has been shown in some cases. For example, infection of *Wolbachia*(+) and *Wolbachia*(-) *Culex quinquefasciatus* colonies with WNV revealed a greater proportion of *Wolbachia*(-) infected mosquitoes developing high virus titers in saliva, which is necessary for virus dissemination and transmission [54]. This observation led to the suggestion that the difference in susceptibility to WNV infection between *Cx*. *quinquefasciatus* and *Culex tarsalis* might be partially explained by the difference in *Wolbachia* infection between these two species, since *Cx*. *tarsalis* is not infected with *Wolbachia* [54].

By applying the metabarcoding approach, we were able to detect several pathogenic species and to confirm several of them by species-specific or genus-specific PCRs. As for *Rickettsia* and *Borrelia* genera, the prevalence in our data set is shared by similar studies in Italy and Europe [55–59]. In addition, our findings show that *Rickettsia* seems to be widespread among residential and migratory birds, while *Borrelia* was detected only in short- and mid-distance migratory birds, suggesting different patterns in its transmission.

The observation of bacterial genera in the metabarcoding results not confirmed by the species-specific or genus-specific PCR tests may be explained by the presence of *Rickettsia*-, *Coxiella*-, and *Francisella*-like symbionts. The primer pairs used for the diagnostic tests were retrieved from published studies on the detection of pathogenic species of these genera. Most likely, these tests fail to detect a symbiont species of the targeted genus. As reported for *Coxiella*, the genetic diversity of symbiont organisms is very high, and little is known about their spread in arthropods, which may explain the discordance between the results of 16S rRNA gene metabarcoding and diagnostic PCRs [60]. Alternatively, it has been shown that some molecular tests that are specific for *C*. *burnetii* also detect *Coxiella*-like bacteria, leading to overestimation of the pathogen species. Indeed, the molecular characterization of bacterial endosymbionts plays a pivotal role in the design of targeted molecular tests for the sole detection of pathogenic species.

Recent studies have shown that *C*. *burnetii* could have originated from a tick-associated ancestor, while the *Francisella*-like endosymbiont of the hard tick *Ambylomma* probably evolved from a pathogenic strain of *Francisella*, indicating that tick endosymbionts can evolve from mammalian pathogens [14,15]. Little is known about these recently uncovered symbionts, perhaps because the research was biased towards the pathogenic species. Such is the case of the *Coxiella* genus, which only has two species (i.e., *burnetii* and *cheraxi*). The majority of studies have described *C*. *burnetii* because most isolates were collected from humans or domestic ruminants during Q fever outbreaks. More information on novel *Coxiella*-like organisms in non-vertebrate species like ticks has been acquired via 16S rRNA gene metabarcoding [14].

Finally, we observed a critical point in the bioinformatics analysis of our data. The first point is the erroneous identification of *E*. *ovina* by the MSR, not confirmed by deeper analysis. This issue may concern the used database, since, as reported in the Illumina manual, the Metagenomics workflow uses an Illumina-curated version of the Greengenes database. The choice of database may also lead to different results in taxa assignment. In our analyses, the SILVA database allowed us to assign RSVs to *Wolbachia*, C. *Midichloria*, and *Arsenophonus*. Since these three genera represent the majority of the microbial community, being symbionts, correct taxonomic assignment is very relevant for these kind of studies. We suggest the use of the SILVA database for future projects investigating arthropod microbiota by 16S rRNA metabarcoding.

Our metabarcoding analysis showed that the microbiota living with (and within) arthropods is complex, closely related to the host species, and that its major component comprises endosymbiont-related species. This approach provides a global overview of the bacteria present in/on ectoparasites collected live from migratory birds. Because it employs a universal primer set for prokaryotic metabarcoding, this approach was also useful for identifying in one shot the genera that include pathogen species. Since the method does not often discriminate beyond the genus level, a second-level, genus- or species-specific investigation was required to confirm the presence of the pathogen species in some samples. Without an overview provided by the metabarcoding method, multiple tests for each pathogen in all the samples would have been needed.

## Supporting information

S1 Fig Map of sampling sites. Sites in Liguria, Latium, and Sicily were located on the islands of Palmaria, Ventotene, and Ustica, respectively. Each of these map tile sets are Stamen Design, under a Creative Commons Attribution (CC BY 3.0) license.
(PDF)

S2 Fig Bar plot of the relative abundance by family of bacteria in *Hippoboscidae* diptera. The abundance is relative only to the family considered and not to the total microbiota.
(PDF)

S3 Fig Bar plot of the relative abundance of *Rickettsiales* in ticks. The abundance is relative only to the *Rickettsiales* family and not to the total microbiota.
(PDF)

S4 Fig Bar plot representing the relative abundance of the RSVs by genus in the *Hippoboscidae* diptera. Abundance is relative to the total microbiota. To improve color-coding readability, RSV numbering is assigned by genus, so that RSV 1 in *Wolbachia* is not the same as RSV 1 in *Arsenophonus*.
(PDF)

S5 Fig Bar plot representing the relative abundance of the RSVs in ticks for the most common genera. Abundance is relative to only the genera considered. To improve color-coding readability, RSV numbering is assigned by genus, so that RSV 1 in *Wolbachia* is not the same as RSV 1 in *Rickettsia*.
(PNG)

S6 Fig Blast output of the reads classified as *E*. *ovina* by MSR and visualized in MEGAN. (PDF)

S1 Table List of bird species caught during ringing sessions and hosting the ectoparasites sampled. (DOC)

S2 Table List of the ectoparasites with their scientific name as obtained by partial sequencing of the COI gene. The number of sampled individuals and the number of pools are reported.
(DOC)

S3 Table Number of reads before and after filtering for each sample and its relative parasite group. (DOC)
