# Supplementary figures and images for: The microbiota of hematophagous ectoparasites collected from migratory birds
Source: PLoS One. 2018 Aug 27;13(8):e0202270. doi: 10.1371/journal.pone.0202270 (PMC6110481; doi:10.1371/journal.pone.0202270)

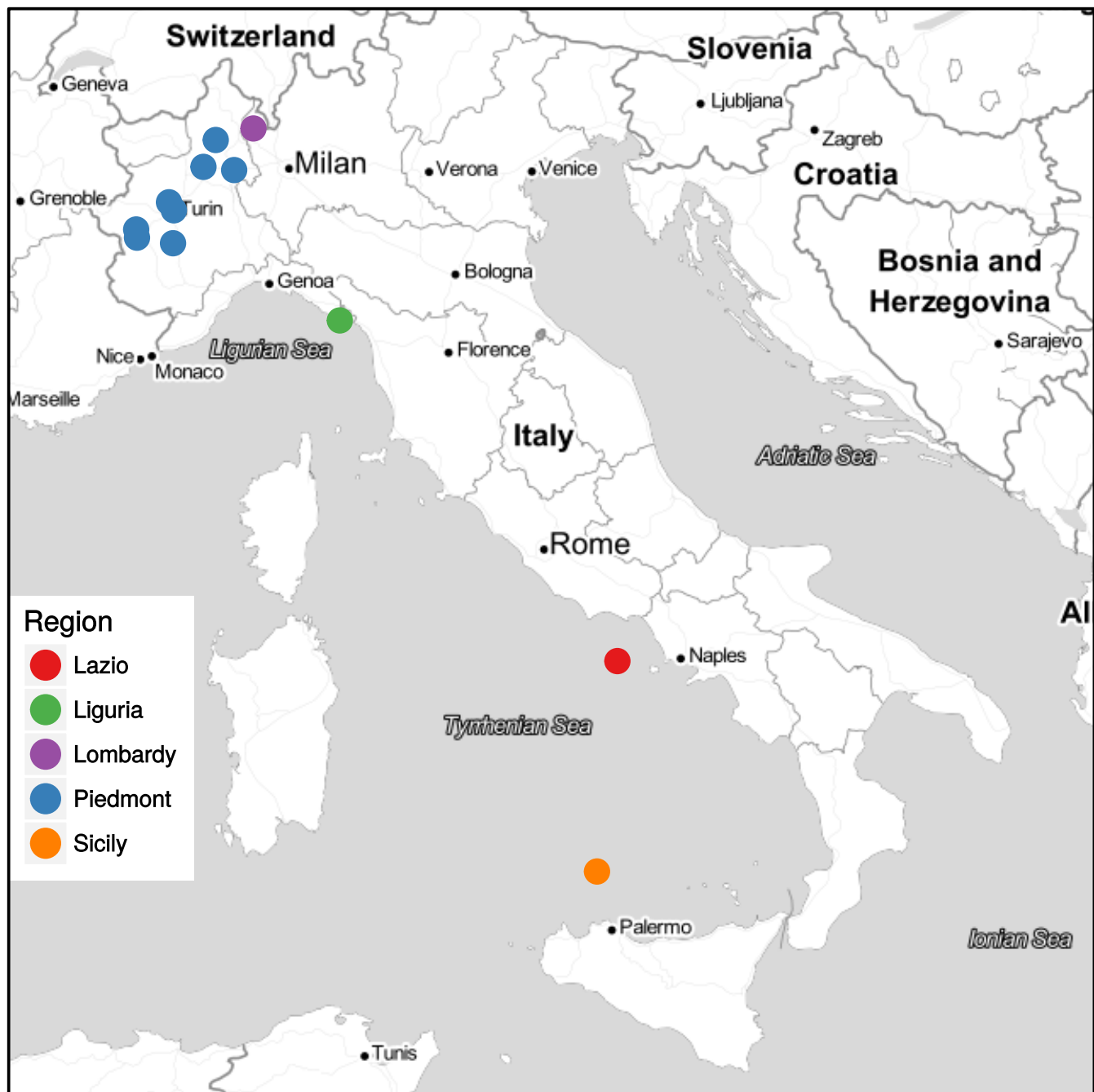

Supplement: S1 Fig — Sites in Liguria, Latium, and Sicily were located on the islands of Palmaria, Ventotene, and Ustica, respectively. Each of these map tile sets are Stamen Design, under a Creative Commons Attribution (CC BY 3.0) license. (PDF) [file pone.0202270.s001.pdf]

# Relative abundance of genera in Hippoboscidae diptera by family

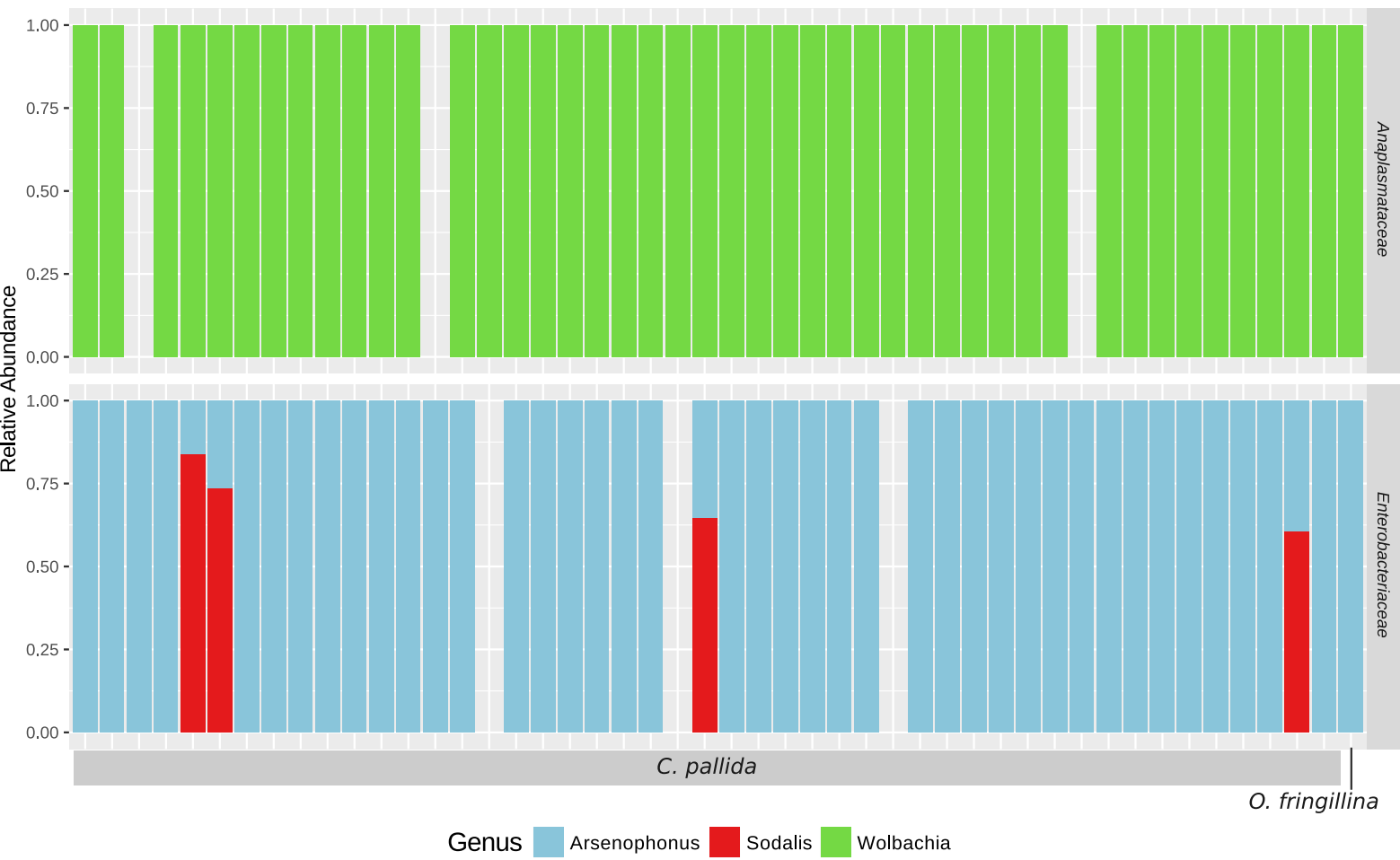

Supplement: S2 Fig — The abundance is relative only to the family considered and not to the total microbiota. (PDF) [file pone.0202270.s002.pdf]

### Relative abundance of *Rickettsiales* from ticks

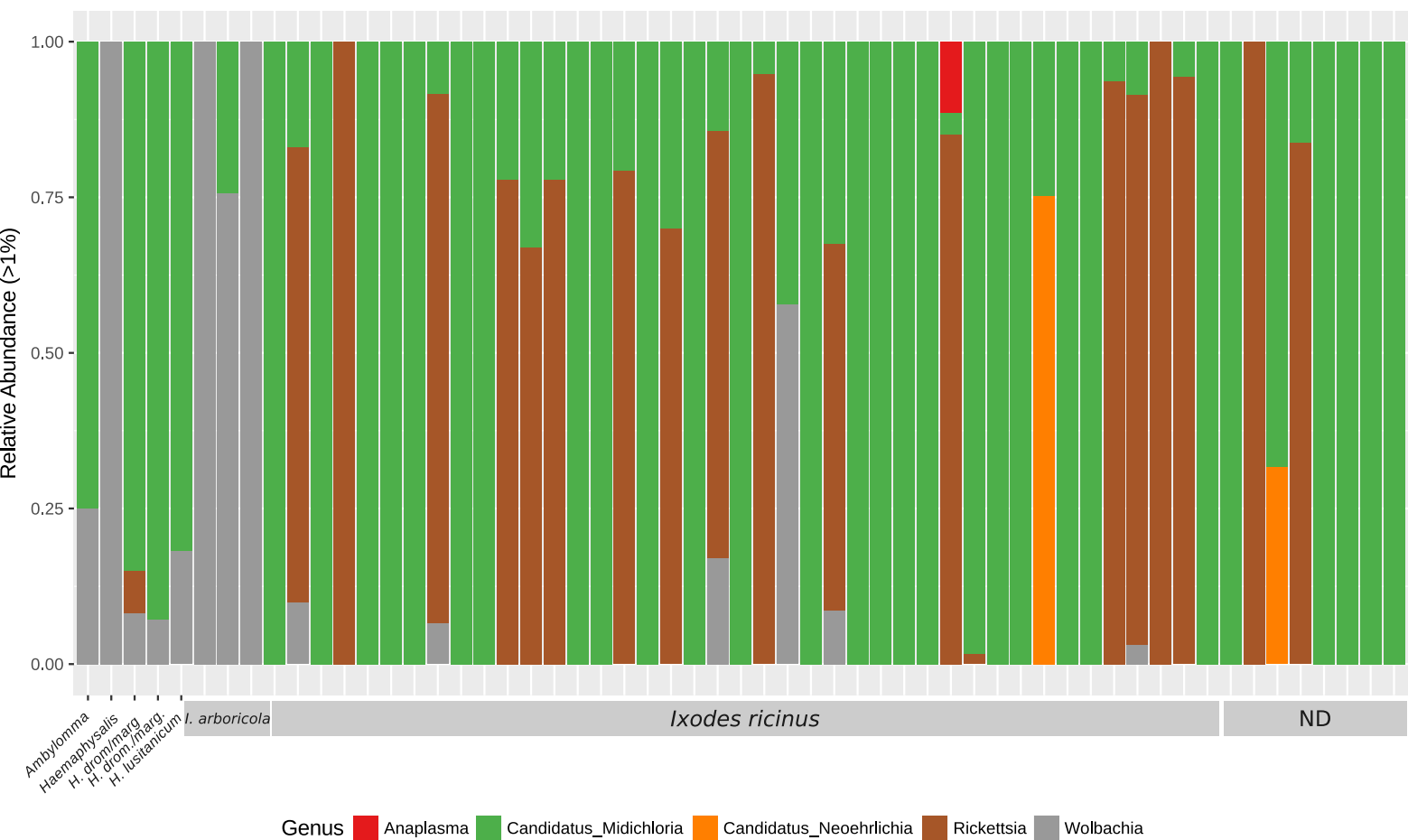

Supplement: S3 Fig — The abundance is relative only to the Rickettsiales family and not to the total microbiota. (PDF) [file pone.0202270.s003.pdf]

### *Hippoboscidae* diptera - RSV distribution by genus

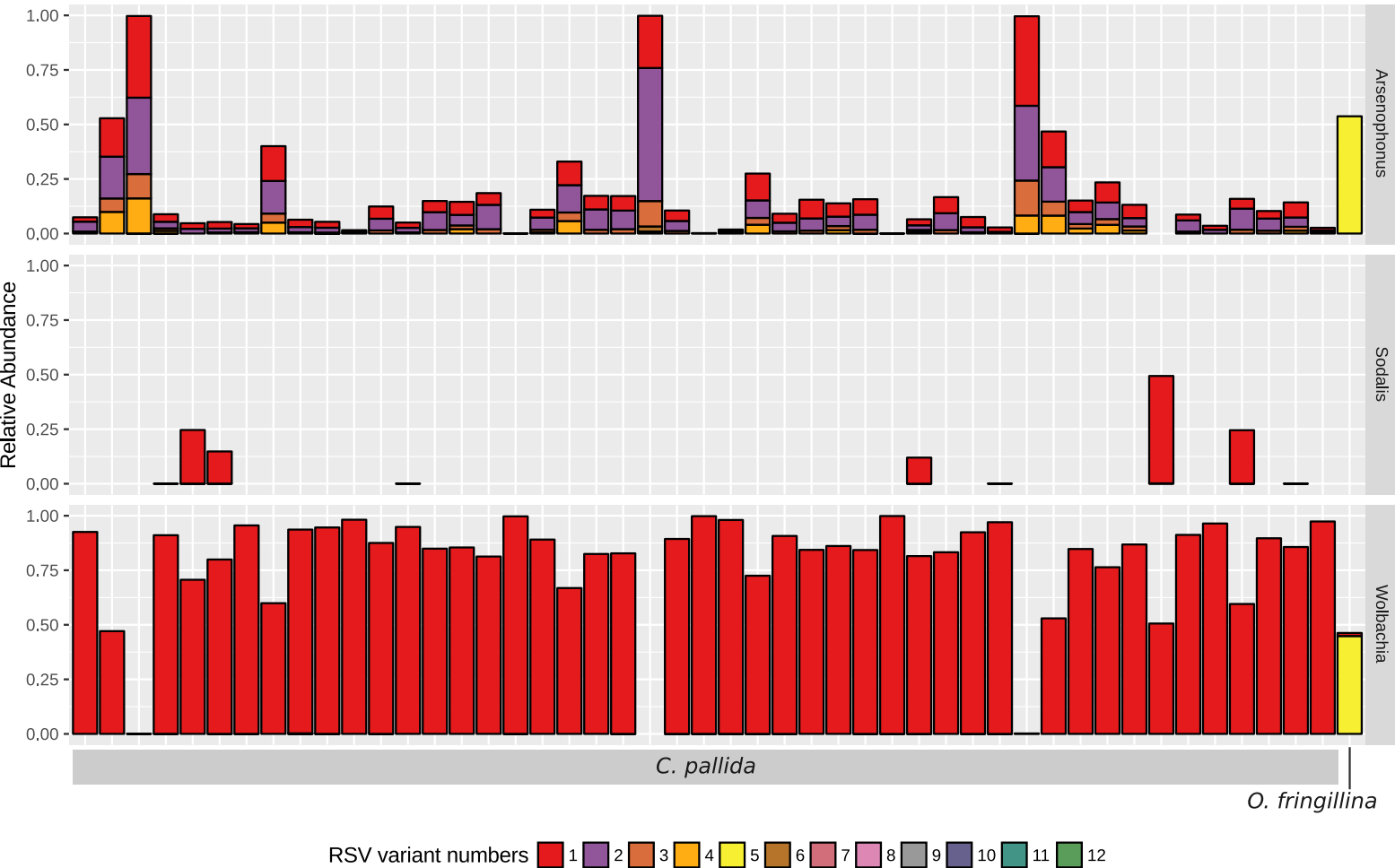

Supplement: S4 Fig — Abundance is relative to the total microbiota. To improve color-coding readability, RSV numbering is assigned by genus, so that RSV 1 in Wolbachia is not the same as RSV 1 in Arsenophonus. (PDF) [file pone.0202270.s004.pdf]

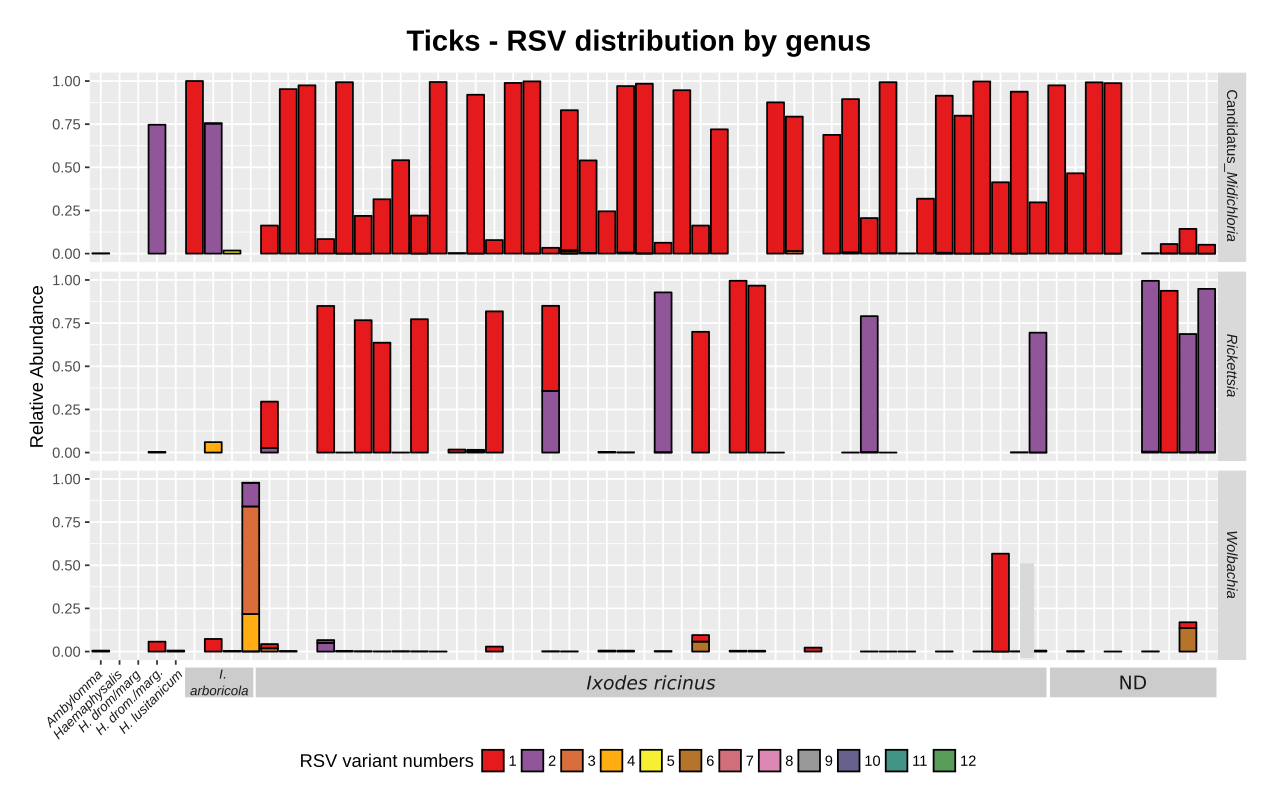

Supplement: S5 Fig — Abundance is relative to only the genera considered. To improve color-coding readability, RSV numbering is assigned by genus, so that RSV 1 in Wolbachia is not the same as RSV 1 in Rickettsia. (PNG) [file pone.0202270.s005.png]

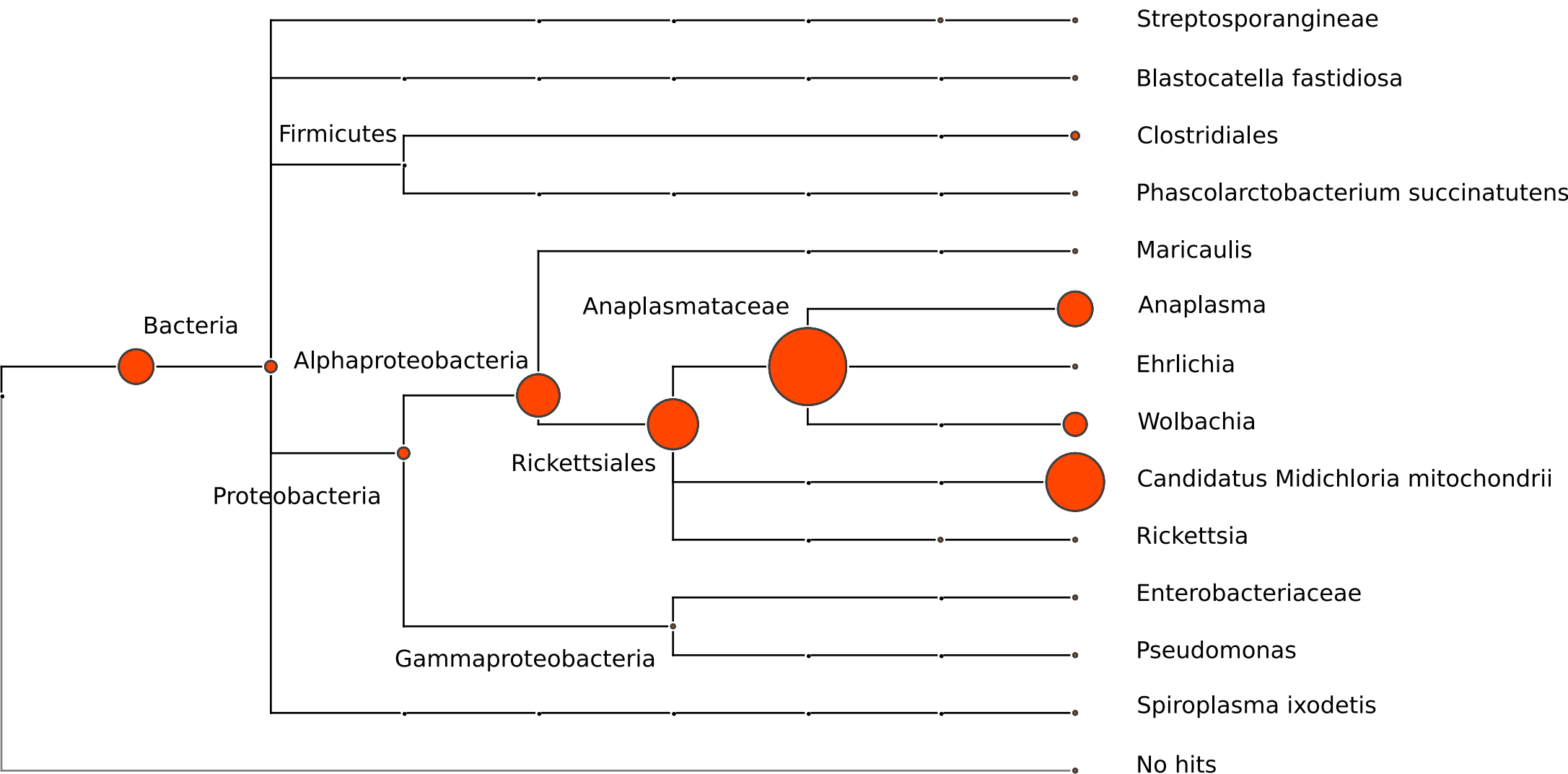

Supplement: S6 Fig — (PDF) [file pone.0202270.s006.pdf]
