# Supplementary material for: The microbiota of hematophagous ectoparasites collected from migratory birds
Source: PLoS One. 2018 Aug 27;13(8):e0202270. doi: 10.1371/journal.pone.0202270 (PMC6110481; doi:10.1371/journal.pone.0202270)
Supplement: S1 Table — (DOC) [file pone.0202270.s007.doc]

**S1 Table. List of bird species caught during the ringing sessions and hosting the sampled ectoparasites.**

| **Host species** | **# catches** |
| --- | --- |
| *Otus scops* | 1 |
| *Acrocephalus scirpaceus* | 1 |
| *Sylvia atricapilla* | 2 |
| *Parus major* | 10 |
| *Aegolius funereus* | 1 |
| *Phoenicurus phoenicurus* | 1 |
| *Oenanthe oenanthe* | 1 |
| *Fringilla coelebs* | 2 |
| *Coccothraustes coccothraustes* | 2 |
| *Anas platyrhynchos* | 2 |
| *Falco tinnunculus* | 1 |
| *Garrulus glandarius* | 1 |
| *Phylloscopus trochilus* | 2 |
| *Turdus merula* | 9 |
| *Prunella modularis* | 1 |
| *Erithacus rubecula* | 5 |
| *Muscicapa striata* | 1 |
| *Buteo buteo* | 1 |
| *Regulus regulus* | 3 |
| *Hirundo rustica* | 1 |
| *Apus apus* | 48 |
| *Saxicola torquatus* | 1 |
| *Troglodytes troglodytes* | 1 |
| *Sylvia communis* | 1 |
| *Sturnus vulgaris* | 1 |
| *Labrus merula* | 1 |
| *Turdus philomelos* | 10 |
| *Turdus iliacus* | 2 |
| ND | 2 |
