# Supplementary material for: The microbiota of hematophagous ectoparasites collected from migratory birds
Source: PLoS One. 2018 Aug 27;13(8):e0202270. doi: 10.1371/journal.pone.0202270 (PMC6110481; doi:10.1371/journal.pone.0202270)
Supplement: S2 Table — The number of sampled individuals and the number of pools are reported. (DOC) [file pone.0202270.s008.doc]

**S2 Table. List of the sampled ectoparasites reported with their scientific name as obtained by the partial sequence of the COI gene.** Aside, the number of sampled individuals, the number of pools and the corresponding group are reported.

| **Parasite species** | **# individuals** | **# pool** | **Group** |
| --- | --- | --- | --- |
| *Crataerina pallida* | 50 | 48 | Hippoboscidae diptera |
| *Ornithomya fringillina* | 2 | 2 | Hippoboscidae diptera |
| *Ambylomma spp.* | 1 | 1 | Ticks |
| *Hyalomma dromedarii/marginatum* | 2 | 2 | Ticks |
| *Hyalomma lusitanicum* | 1 | 1 | Ticks |
| *Hyalomma spp* | 4 | 1 | Ticks |
| *Ixodes arboricola* | 5 | 4 | Ticks |
| *Ixodes ricinus* | 88 | 45 | Ticks |
| *Anatoecus dentatus* | 6 | 1 | Other arthropods |
| *Anystis* | 6 | 1 | Other arthropods |
| *Aphidiinae spp.* | 1 | 1 | Other arthropods |
| *Colpocephalum turbinatum* | 2 | 1 | Other arthropods |
| *Haemaphysalis sp* | 1 | 1 | Other arthropods |
| *Lucilia caesar* | 11 | 1 | Other arthropods |
| ND | 14 | 10 |  |
| **Total** | **194** | **120** |  |
