# Supplementary material for: The microbiota of hematophagous ectoparasites collected from migratory birds
Source: PLoS One. 2018 Aug 27;13(8):e0202270. doi: 10.1371/journal.pone.0202270 (PMC6110481; doi:10.1371/journal.pone.0202270)
Supplement: S3 Table — (DOC) [file pone.0202270.s009.doc]

**S3 Table. Number of reads before and after filtering for each sample and its relative parasite group.**

| **Sample name** | **Total reads** | **Total reads after filtering** | **Parasite species** | **Parassite type** |
| --- | --- | --- | --- | --- |
| 12DXC001 | 265,732 | 84,334 | *Crataerina pallida* | Hippoboscidae diptera |
| 13DRM002 | 376,320 | 95,294 | *Crataerina pallida* | Hippoboscidae diptera |
| 13DRM003 | 1,316,978 | 392,714 | *Crataerina pallida* | Hippoboscidae diptera |
| 13DRM004 | 701,952 | 199,582 | *Crataerina pallida* | Hippoboscidae diptera |
| 13DRM005 | 363,960 | 82,556 | *Crataerina pallida* | Hippoboscidae diptera |
| 13DRM007 | 462,858 | 134,122 | *Crataerina pallida* | Hippoboscidae diptera |
| 13DRM009 | 305,626 | 140,232 | *Crataerina pallida* | Hippoboscidae diptera |
| 13DRM010 | 495,868 | 185,686 | *Crataerina pallida* | Hippoboscidae diptera |
| 13DRM011 | 455,794 | 202,652 | *Crataerina pallida* | Hippoboscidae diptera |
| 13DRM012 | 2,412,330 | 597,326 | *Crataerina pallida* | Hippoboscidae diptera |
| 13DRM013 | 241,608 | 93,958 | *Crataerina pallida* | Hippoboscidae diptera |
| 13DRM014 | 366,006 | 80,932 | *Crataerina pallida* | Hippoboscidae diptera |
| 13DRM015 | 514,684 | 179,964 | *Crataerina pallida* | Hippoboscidae diptera |
| 13DRM030 | 296,050 | 72,038 | *Crataerina pallida* | Hippoboscidae diptera |
| 13DRM031 | 384,136 | 96,910 | *Crataerina pallida* | Hippoboscidae diptera |
| 13DRM032 | 789,750 | 213,098 | *Crataerina pallida* | Hippoboscidae diptera |
| 13DRM033 | 447,046 | 143,512 | *Crataerina pallida* | Hippoboscidae diptera |
| 13DRM034 | 546,188 | 210,020 | *Crataerina pallida* | Hippoboscidae diptera |
| 13DRM035 | 231,378 | 75,980 | *Crataerina pallida* | Hippoboscidae diptera |
| 13DRM036 | 289,328 | 92,858 | *Crataerina pallida* | Hippoboscidae diptera |
| 13DRM037 | 619,310 | 182,518 | *Crataerina pallida* | Hippoboscidae diptera |
| 13DRP016 | 319,760 | 13,654 | *Crataerina pallida* | Hippoboscidae diptera |
| 13DRP017 | 323,508 | 126,062 | *Crataerina pallida* | Hippoboscidae diptera |
| 13DRP018 | 325,118 | 83,428 | *Crataerina pallida* | Hippoboscidae diptera |
| 13DRP019 | 519,820 | 167,984 | *Crataerina pallida* | Hippoboscidae diptera |
| 13DRP020 | 466,154 | 138,592 | *Crataerina pallida* | Hippoboscidae diptera |
| 13DRP021 | 444,312 | 126,966 | *Crataerina pallida* | Hippoboscidae diptera |
| 13DRP022 | 199,700 | 35,004 | *Crataerina pallida* | Hippoboscidae diptera |
| 13DRP023 | 265,230 | 65,712 | *Crataerina pallida* | Hippoboscidae diptera |
| 13DRP024 | 283,604 | 81,442 | *Crataerina pallida* | Hippoboscidae diptera |
| 13DRP025 | 389,844 | 126,630 | *Crataerina pallida* | Hippoboscidae diptera |
| 13DRP026 | 570,874 | 186,070 | *Crataerina pallida* | Hippoboscidae diptera |
| 13DRP027 | 191,070 | 56,358 | *Crataerina pallida* | Hippoboscidae diptera |
| 13DRP028 | 477,340 | 152,006 | *Crataerina pallida* | Hippoboscidae diptera |
| 13DRP029 | 230,428 | 87,554 | *Crataerina pallida* | Hippoboscidae diptera |
| 14DRM053 | 363,386 | 95,234 | *Crataerina pallida* | Hippoboscidae diptera |
| 14DRM054 | 429,186 | 83,460 | *Crataerina pallida* | Hippoboscidae diptera |
| 14DRM055 | 186,696 | 61,704 | *Crataerina pallida* | Hippoboscidae diptera |
| 14DRM056 | 363,966 | 94,690 | *Crataerina pallida* | Hippoboscidae diptera |
| 14DRM057 | 242,972 | 73,888 | *Crataerina pallida* | Hippoboscidae diptera |
| 14DRM059 | 848,670 | 206,070 | *Crataerina pallida* | Hippoboscidae diptera |
| 14DRM060 | 421,286 | 134,106 | *Crataerina pallida* | Hippoboscidae diptera |
| 14DRM061 | 206,134 | 60,224 | *Crataerina pallida* | Hippoboscidae diptera |
| 14DRM062 | 775,122 | 200,764 | *Crataerina pallida* | Hippoboscidae diptera |
| 14DRM063 | 294,342 | 45,684 | *Crataerina pallida* | Hippoboscidae diptera |
| 14DRP051 | 271,748 | 74,226 | *Crataerina pallida* | Hippoboscidae diptera |
| 14DRP052 | 348,322 | 112,612 | *Crataerina pallida* | Hippoboscidae diptera |
| 14DRP104 | 309,124 | 57,810 | *Ornithomya fringillina* | Hippoboscidae diptera |
| 06ZLV040 | 97,554 | 14,386 | *Ixodes arboricola* | Tick |
| 08ZCB038 | 8,442 | 836 | *Ixodes arboricola* | Tick |
| 08ZPB039 | 817,698 | 61,034 | ND | Tick |
| 13ZAU090 | 252,048 | 53,990 | *Hyalomma dromedarii/marginatum* | Tick |
| 13ZCT038 | 101,832 | 24,118 | *Ixodes arboricola* | Tick |
| 13ZRU089 | 1,782 | 34 | *Ixodes ricinus* | Tick |
| 13ZSU088 | 458,450 | 155,692 | *Hyalomma dromedarii/marginatum* | Tick |
| 13ZXU091 | 639,400 | 86,356 | *Hyalomma lusitanicum* | Tick |
| 14ECC040 | 1,357,174 | 558,862 | *Ixodes arboricola* | Tick |
| 14ZCB085 | 49,620 | 12,582 | *Ixodes ricinus* | Tick |
| 14ZCB087 | 150,308 | 44,896 | *Ixodes ricinus* | Tick |
| 14ZCV046 | 179,740 | 66,378 | *Ixodes ricinus* | Tick |
| 14ZCV047 | 36,780 | 3,956 | *Ixodes ricinus* | Tick |
| 14ZCV066 | 518,240 | 209,274 | *Ixodes ricinus* | Tick |
| 14ZCV067 | 666,828 | 141,316 | *Ixodes ricinus* | Tick |
| 14ZCV068 | 556,432 | 70,926 | *Ixodes ricinus* | Tick |
| 14ZCV069 | 251,160 | 71,894 | ND | Tick |
| 14ZCV070 | 602,296 | 151,898 | *Ixodes ricinus* | Tick |
| 14ZCV071 | 81,860 | 36,748 | *Ixodes ricinus* | Tick |
| 14ZCV072 | 309,764 | 25,424 | ND | Tick |
| 14ZFP096 | 76,416 | 29,148 | *Ixodes ricinus* | Tick |
| 14ZFP105 | 493,948 | 108,282 | *Ixodes ricinus* | Tick |
| 14ZFP111 | 95,520 | 41,902 | *Ixodes ricinus* | Tick |
| 14ZFP112 | 273,612 | 63,424 | ND | Tick |
| 14ZGP095 | 229,640 | 55,258 | *Ixodes ricinus* | Tick |
| 14ZLB086 | 505,588 | 115,472 | *Ixodes ricinus* | Tick |
| 14ZMC082 | 299,090 | 56,466 | ND | Tick |
| 14ZMP099 | 256,030 | 50,402 | *Ixodes ricinus* | Tick |
| 14ZMP101 | 222,322 | 31,294 | *Ixodes ricinus* | Tick |
| 14ZMP102 | 864,616 | 227,572 | *Ixodes ricinus* | Tick |
| 14ZMP103 | 270,800 | 35,334 | *Ixodes ricinus* | Tick |
| 14ZMV042 | 78,142 | 16,954 | *Ixodes ricinus* | Tick |
| 14ZMV044a | 490,756 | 167,672 | *Ixodes ricinus* | Tick |
| 14ZMV044b | 499,406 | 61,148 | *Ixodes ricinus* | Tick |
| 14ZMV044c | 299,076 | 78,300 | *Ixodes ricinus* | Tick |
| 14ZMV045a | 74,528 | 23,338 | ND | Tick |
| 14ZMV045b | 753,656 | 124,520 | *Ixodes ricinus* | Tick |
| 14ZMV045c | 81,692 | 26,108 | *Ixodes ricinus* | Tick |
| 14ZMV065 | 724,084 | 114,358 | *Haemaphysalis sp* | Tick |
| 14ZPB084 | 99,616 | 28,100 | *Ixodes ricinus* | Tick |
| 14ZPC073 | 551,232 | 53,200 | *Ixodes ricinus* | Tick |
| 14ZPC074 | 592,456 | 157,594 | *Ixodes ricinus* | Tick |
| 14ZPC075 | 610,210 | 235,460 | ND | Tick |
| 14ZPP049 | 132,988 | 26,238 | *Ambylomma spp.* | Tick |
| 14ZPP094 | 161,210 | 10,624 | ND | Tick |
| 14ZRP092 | 10 | 0 | ND | Tick |
| 14ZRP107 | 591,822 | 143,306 | *Ornithomya fringillina* | Tick |
| 14ZSV048 | 693,756 | 105,372 | *Ixodes ricinus* | Tick |
| 14ZSV080 | 793,158 | 180,416 | *Ixodes ricinus* | Tick |
| 14ZTB083 | 96,298 | 35,980 | *Ixodes ricinus* | Tick |
| 14ZTC076 | 452,542 | 211,422 | *Ixodes ricinus* | Tick |
| 14ZTC077 | 224,274 | 64,338 | *Ixodes ricinus* | Tick |
| 14ZTC078 | 142,438 | 50,502 | *Ixodes ricinus* | Tick |
| 14ZTP093 | 317,200 | 47,474 | *Ixodes ricinus* | Tick |
| 14ZTP097 | 371,648 | 146,780 | *Ixodes ricinus* | Tick |
| 14ZTP098 | 201,346 | 82,290 | *Ixodes ricinus* | Tick |
| 14ZTP100 | 1,270 | 6 | *Ixodes ricinus* | Tick |
| 14ZTP108 | 800,336 | 225,502 | *Ixodes ricinus* | Tick |
| 14ZTP110 | 296,602 | 38,640 | *Ixodes ricinus* | Tick |
| 14ZTV079 | 482,240 | 130,112 | *Ixodes ricinus* | Tick |
| 14ZTV081 | 149,934 | 24,380 | *Ixodes ricinus* | Tick |
| 08MGR041 | 426 | 0 | ND | Other ectoparasite |
| 13PRM008 | 184,336 | 9,226 | ND | Other ectoparasite |
| 14MCP106 | 632,740 | 264,490 | *Anystis* | Other ectoparasite |
| 14MGR050 | 29,402 | 1,598 | *Anatoecus dentatus* | Other ectoparasite |
| 14MGR058 | 385,700 | 101,956 | *Lucilia caesar* | Other ectoparasite |
| 14MPT064 | 453,370 | 75,156 | *Colpocephalum turbinatum* | Other ectoparasite |
| 14MTP109 | 160,508 | 34,268 | *Aphidiinae spp.* | Other ectoparasite |
